# Effect of Soil Nutrient on Production and Diversity of Volatile Terpenoids from Plants

**DOI:** 10.2174/157340712799828188

**Published:** 2012-01

**Authors:** E Ormeño, C Fernandez

**Affiliations:** Aix-Marseille University – Equipe Diversité et Fonctionnement : des Molécules aux Ecosystèmes - Institut Méditerranéen de Biodiversité et d’Ecologie (IMBE) (marine et continentale) UMR 7263 CNRS. Centre St Charles, Case 4, 13331 Marseille Cedex 03, France

**Keywords:** Emissions, storage, isoprene, monoterpenoids, chemical defenses, nitrogen, phosphorus, interaction.

## Abstract

Terpenoid production (emission and storage) within foliage plays direct and indirect defensive and protective functions for the plant, mediates complex trophic relationships and controls the oxidation capacity of the atmosphere. Both biotic and abiotic conditions alter terpenoid production, with herbivory, light and temperature effects being reasonably well understood. In this manuscript, the state of the science about nutrient effect on terpenoid production is reviewed. The focus is on isoprene emissions and mono- and sesquiterpenoid emissions and concentrations according to fertilizing treatments and their potential interaction with other environmental factors. Ecological, physiological, biochemical and biophysical hypothesis formulated over research investigations are exposed and several points are highlighted as future research perspectives which could help to elucidate the apparent contrasting results.

## 1 INTRODUCTION

Plants collectively produce thousands of biogenic volatile organic compounds (BVOCs). Terpenoids, sometimes referred to as isoprenoids, are the most diverse class of BVOCs. They are low weight hydrocarbons, mostly cyclic although acyclic forms also exist (e.g. myrcene), formed at least by carbon and hydrogen, but they may also present oxygen. Other BVOCs are benzenics, N- and S- containing compounds. According to the number of C_5_ units, terpenoids are defined as hemiterpenoids, monoterpenoids, and sesquiterpenoids, with 5, 10, and 15 carbon skeletons respectively. As the number of carbon units increases, their chemical diversity increases too. Thus, hemiterpenoids are mostly represented by isoprene and methyl butenol, around 1000 different structures have been reported for monoterpenoids, with limonene and a-pinene being the most common, and close to 5000 sesquiterpenoids have been detected in plants, the most universal being b-caryophyllene [1]. 

The terms “storage”, “emission”, and “production” dominate in literature studies, but are not necessarily always used with the same meaning. These terms will be used throughout this review and hence need to be clarified. The term “BVOC production” is large and refers to both what is emitted through the leaf and what is stored within it. “BVOC storage” - also referred to as “specifically stored BVOCs” - accounts for the accumulation of important amounts of BVOCs (µg.g_DM_^-1^) in specialized storage structures typically classified as secretory cells, secretory cavities (commonly referred to as oil glands in *Citrus* sp., and as resin canals in* Pinus *sp.), ducts (commonly referred to as oil ducts), glandular trichomes, and glands. BVOCs are either supplied to the structures by vascular tissues or synthesized by their constituent cells. These permanent and large reservoirs of BVOCs are mainly filled up with mono-and sesquiterpenoids which account for a potential source of BVOC emissions, even if BVOC synthesis is inactive due to some external stress factor (e.g. drought). Other compounds, such as isoprene and methyl butanol, as well as green leaf volatiles (e.g. induced C_6_–aldehydes, C_6_-alcohols, and their acetates released under biotic pressures), are never specifically stored, probably due to their high volatility. Other species do not present such specific reservoirs. Instead, they synthesize BVOC-precursors in the chloroplasts and cytosol of mesophyll cells. As a result, only temporary pools of these *de novo* formed BVOCs - also referred to as non-specific storage structures - may be present within the intercellular spaces and make possible that BVOC emissions occur for minutes (isoprene) or hours (monoterpenoids) even if BVOC synthesis is stopped (e.g. absence of light). Although this dual scheme (storing and non-storing species) is massively used and accepted, investigations performed during the last decade by means of C-13 have evidenced that specific and non-specific forms of BVOC storage occur in the same species and that despite the presence of specific BVOC reservoirs, mono- and sesquiterpenoids may also be the novo synthesized. Knowledge of the presence of these structures within a species may help to understand how abiotic factors affect terpenoid production. In non-storing species (e.g. *Quercus ilex* L., *Populus* sp.), terpenoid emissions often feature stronger and faster short-term responses to environmental factors due to the absence of terpenoid reservoirs that buffer the direct influence of the environment [2,3].

BVOC emission and storage allow plants to withstand numerous abiotic and biotic stress conditions and mediate ecological interactions with the biotic environment [1]. Plants are not passive victims against herbivory since the important amounts of terpenoids specifically stored within plant tissues may act as antiherbivore chemical defences which make leaf consumption toxic for herbivores leading them to change their dietary habits and reducing the success of invading herbivores and pathogens. Complex mixtures of BVOC emissions also play an important role in the recruitment of the carnivorous natural enemies of herbivores [4]. Leaf emissions of BVOCs, especially isoprene, protect the leaf cell against short episodes of heat stress [5]. Terpenoids are highly reactive gases, and are emitted in such large quantities from the biosphere that substantially affect the oxidizing potential of the atmosphere and intervene in ozone (O_3_) and some aerosol formation [6].

Since BVOC emissions are highly sensitive to abiotic factors, ecological roles of BVOCs may be endangered or modified by environmental changes, including global-change related phenomena. While the effect of climate-related factors is well documented, soil nutrient impact on BVOC fluxes has received relatively little attention [7]. Both excessive soil fertilization and nutrient starvation in abandoned lands are however major problems nowadays. The aim of this review is to summarize investigations into the impact of soil nutrients applied through different fertilizers on BVOC plant production, with a special focus on leaf emission and storage of terpenoids. 

## 2 INFLUENCE OF NUTRIENTS ON TEROENOID PRODUCTION

### 2.1 Biochemical and Biophysical Explanations

Nitrogen could promote terpenoid emissions by promoting electron transport rate and leaf photosynthesis which provide ATP requirements and carbon substrate availability for isoprene synthesis. Nitrogen is expected to favor terpenoid production, especially isoprene emissions, and mono- and sesquiterpenoid emissions in non-storing species which often feature stronger and faster short-term responses to environmental factors due to the absence of terpenoid reservoirs that buffer the direct influence of the environment [2,3]. In storing species, although specific storage structures may uncouple BVOC emissions from photosynthesis, all carbon-based secondary metabolites ultimately depend on CO_2_ fixation and, as a result, a relationship between nitrogen and stored terpenoids can also occur. This is supported by previous work [8] that reported a direct relationship between photosynthetic carbon products like glyceraldehyde-3-phosphate or pyruvate, and terpenoids biosynthesis as well as by studies were positive relationships between leaf or soil nitrogen and terpenoid concentration in leaves has been found [9,10].

Phosphorus is expected to influence terpenoid production since terpenoid precursors (IPP: isopentenyl diphosphate and DMAPP: Dimethylallyl pyrophosphate) contain high-energy phosphate bonds and phosphorus is a key component of ATP and NADPH which are required for terpenoid synthesis. Niinemets *et al*. (2002) [11] estimated that *Quercus coccifera* L. requires 28 moles of NADPH and 40 moles of ATP to synthesize monoterpenoids. Phosphorus could hence be a key limiting nutrient involved in terpenoid emission and storage. 

Isoprene emission could also be related to phosphorus availability since deprivation of this macronutrient degrades cell membranes, part of which seems to be compensated by greater isoprene emissions. In particular, phosphorus starvation reduces the amounts of phospholipids that form the bilayer in cell membranes, crucial for life in all organisms since it separates the interior of cells from their environment [12]). Siwko *et al*. (2007) [13] demonstrated that under heat stress, this bilayer degradation is reduced by isoprene emissions, which ensure the stability of the bilayer at a concentration of 20 mol % isoprene (16 isoprene molecules per 64 lipid molecules). The effect of this isoprene addition on the membrane is equivalent to a reduction in temperature of 10 K, rising to a reduction of 30 K at 43 mol % isoprene. Likewise, the phospholipid bilayer instability under phosphorus starvation could be copped by greater isoprene emission. 

### 2.2 What Ecological Theories Anticipate 

In the early 1980s, attention began to be focused on the role of nutrient resource availability in terms of the costs and benefits of producing carbon-based metabolites such as terpenoids. This attention resulted in 2 resource allocation theories used for predicting allocation of carbon and nutrient resources for the production of carbon-based defense compounds, especially phenolics and terpenoids. The carbon-nutrient balance hypothesis (CNBH) presumes that carbon and nutrient availability in the plant environment determines the production of metabolites. When nutrients, especially nitrogen, are highly scarce, a plant will allocate proportionately more of an abundant resource, such as carbon, to the acquisition of the scarce resource and to the synthesis of defensive compounds [14]. This was based on the observation that limited nutrient resources curtailed plant growth, rather than photosynthesis [15], resulting in an excess of carbohydrates. Under such conditions, the CNBH asserts that the excess of carbohydrates is not used for growth but provides, instead, an additional substrate to synthesize defense secondary metabolites. This theory considers that carbon-based defense compounds have no cost since they do not directly compete with growth, because their synthesis is achieved through an excess of carbohydrates. 

The growth differentiation balance hypothesis (GDBH), also referred to as “excess carbon hypothesis”, assumes that there are 3 types of balance between growth and terpenoid production. Whenever all required resources for growth are available, that is under soils rich in nutrient resources, the theory prescribes that growth (e.g. cell division, biomass production), will be favored over differentiation (e.g. cell maturation and production of defensive compounds) [16-18]. As nitrogen becomes scarcer and not optimal, differentiation will predominate, and consequently terpenoid accumulation or emission will increase at the expense of growth, since the plant allocates proportionately more of an abundant resource, such as carbon, to the acquisition of the scarce resource and to the synthesis of defensive compounds. Finally, under limiting nutrient conditions, both primary and secondary metabolisms are at their lowest levels.

### 2.3 Controversial Between Theory and Practice

Most studies focused on isoprene emissions under fertilization treatments, indicate that there is a positive dependence with nutrients, although this has mainly been demonstrated for nitrogen (Table **1**). High leaf nitrogen concentrations favor photosynthetic activity which is mostly coupled to isoprene emissions. This second relationship occurs because isoprene synthesis relies on the availability of recently assimilated carbon which is used to form photosynthetic metabolites shunted into the methylerythritol pyrophosphate pathway (MEP), whereby isoprene is formed [19]. Positive correlations between isoprene emission rates and leaf nitrogen concentration also support the existence of a mechanism linking leaf nitrogen status and isoprene synthase activity [20]. While most studies indicate a positive effect of leaf nitrogen on leaf isoprene emissions, the impact of phosphorus is less certain. In the herbaceous species *Phragmites australis* grow under different phosphorus levels, it was shown that isoprene emissions were substantially lower under rich phosphorus levels despite increase in photosynthesis rates [21]. Facing this surprising result, authors argued that isoprene was probably limited by factors other than photosynthetic intermediate availability or by energetic (ATP) requirements under high phosphorus levels.

Influence of fertilization on terpenoid storage is by far more controversial (Table **1**). For example, under nitrogen supply, terpenoid content in* Pinus sylvestris* needles was promoted [22], unchanged [23] or disfavored in mature needles [24]. In *Eucalyptus *sp., which stores terpenoids in oil glands beneath the leaf surface, high-fertilization favored terpenoid concentration under nursery conditions while it remained invariable in field conditions [25]. 

Researchers have put forward several hypotheses to explain these seemingly paradoxical effects of nutrient influence on terpenoid storage. Bjorkman *et al*. (1991) [26] monitored changes in nitrogen and resin acid concentrations in needles of young Scots pine trees fertilized with ammonium nitrate (NH_4_NO_3_) over 3 years. They noted that both nitrogen and resin acid concentrations are increased in fertilized trees. The authors suggested that soils rich in nitrogen favor resin duct formation in needles and that resin acid concentrations in needles are directly limited by the size of these storage structures rather than by the substrate for resin acid synthesis. In other words, they suggest that nitrogen influences the terpenoid storage through leaf morphological rather than biochemical changes. This assumption is bolstered by evidence of a positive correlation between terpenoid abundance and the density of storage structures [27] as well as a positive relationship between terpenoid content and leaf thickness, measured as LMA and resulting from increasing nitrogen concentrations [28] (Fig. (**1**)). These results evidence the importance of making the difference between the anatomical (number and size of secretion structures) and the physiological and biochemical factors (enzyme kinetic, precursor availability, photosynthesis) in order to clarify any modification in terpenoid production due to soil nutrients. However, in a later study, whereas nitrogen fertilization increased the number of resin ducts in mature needles of *Pinus sylvestris *L., the concentration of terpenoids decreased [24]. This later study suggests that even if plants may feature a higher number of storage structures under richer soils, these structures still need to be filled up in order to notice a significant effect of nitrogen on terpenoid compound concentration. The incapacity of plants to fill them up as their number increase may be due to the high cost to produce terpenoids per gram, as compared to other secondary metabolites [29]. This later author, reviewing several decades of research, proposes that the cost of producing different terpenoid mixtures and the cost of maintaining different pool sizes, could explain why terpenoid dependency on soil nutrients is species-specific. 

A different explanation was given by Lerdau *et al*. (1995) [9] in the only work that has attempted to study the terpenoid production variation (emission and content) according to nitrogen fertilization over the phenological cycle. The authors found that *Pseudotsuga-Menziesii* Mirb. presented higher needle monoterpenoid concentrations and emission rates under the lowest levels of nitrogen fertilization on condition when plants exhibited a leaf expansion state, exclusively. For the rest of the seasonal cycle this species exhibited the opposite behavior. In other words, the predictions of the resource availability theories were not consistent with experimental results during the most important part of the phenological cycle of this species. Lately, Lerdau *et al*. (1997) [30], in an attempt to salvage the GDBH and CNBH, restricted their scope by suggesting that these theories are useful in order to predict terpenoid changes in annual species (e.g. *Heterotheca-Subaxillaris* [31]) which spend most of their lives growing but fail to predict terpenoid changes in woody species in part because do not consider plant phenology. 

Muzika and Pregitzer 1992 [32] stated that soil nitrogen availability has little to no effect on terpenoid storage since, unlike phenolic compounds, terpenoids are not synthesized through the shikimic acid pathway as aromatic amino acids. As a result, their storage does not rely on competition for nitrogen resources, a hypothesis that has been supported by the existence of correlations between phenolic compound and aromatic amino acid concentrations [33]. Even if there has been a clear attempt to restrict this theory, as well as the GDBH. These theories to phenolic compounds alone, Koricheva (2002) [34] simply concluded that there is a more fundamental problem: the failure of the CNBH as an explanatory tool of the mechanisms that underline the nutrient effect on carbon-based secondary metabolites because a negative effect of nutrient supply on carbon-based secondary compounds could just occur due to a dilution phenomenon since nutrients favor biomass production. In addition, the author indicates that the enzymes responsible for the synthesis of secondary compounds can have sucrose-responsive promoter elements, and therefore react to an increased substrate supply with enhanced rates of transcription. Although severely criticized in the last decade, both the GDBH and CNBH may still help to explain changes in terpenoid concentration due to nutrient availability changes at a time scale of years [35]. 

Other authors recently reported that concentration and proﬁle of constitutive and hervivore-induced monoterpenoid and sesquiterpenoid concentrations in *Pinus pinaster* Aiton,were not affected by phosphorus limitation [36]. They argued that terpenoid homeostasis is probably a way of controlling terpenoid emissions which otherwise would carry airborne information to insects about the nutritional status of the plant. 

Much of the apparently contrasting results could probably be clarified if leaf morpho-anatomy analysis (leaf storage structure size, number) were coupled with cell biochemistry (enzyme kinetic, precursor availability) and, physiology (photosynthesis) in leaves (Fig. (**1**)). Grouping species according to their storage structures (e.g. resin canals in *Pinaceae* sp., and trichomes in *Lamiaceae* sp.), could also bring some light into the terpenoid production pattern according to fertilization treatments. More importantly, it is often ignored that the GDBH suggests the occurrence of 3 different balances between primary and secondary metabolisms under low, intermediate and high nutrient resources respectively, while knowledge of the actual optimal, intermediate and limiting nutrient conditions of a given species are rarely examined. As a result, the GDBH often fails to explain the scientific results and studies with similar fertilization rates result in different growth – terpenoid balances in different species. 

## 3 INTERACTION BETWEEN FERTILIZATION TREATMENTS AND OTHER ENVIRONMENTAL FACTORS

Substantial efforts have been put into defining the impact of different fertilizers at different rates. The influence of fertilizing treatments is most often tackled considering a single type of fertilizer supplied into soil at different rates, typically 3 or 4 rates, while irrigation is regularly applied as required by the plant. More rarely, protocols consider the combined action of different fertilizers and their interaction with either abiotic and biotic factors and the resulting effects in terpenoid metabolism (Fig. (**2**)). 

Kandeel *et al.* [37] focused on the effect of inorganic and organic nitrogen fertilizers and their combinations on yield and oil composition of basil. They showed that when combined, nitrogen supply increased oil yield (mainly composed by terpenoid-like compounds) compared to plants fertilized with inorganic nitrogen alone. The mixture of inorganic and organic nitrogen also increased or decreased the concentration of different BVOCs contained within the essential oil. Other authors [38] reported a high essential oil yield in *Foeniculum vulgare* Mill. when using a mixture of 50% of the recommended dosage of NPK and a biofertilizer (inoculation of *Azotobacter chroococcum, Azospirillum liboferum*, and *Bacillus megatherium*). A low yield was obtained when only 50 % of the inorganic fertilizer was applied without inoculation. This is due to some free-living nitrogen-fixing bacteria into the soil presenting the ability to fix nitrogen and release phytohormones similar to gibberellic acid and indole acetic acid, which stimulate plant growth, nutrient absorption and photosynthesis.

In a study performed in the Mediterranean area under garden conditions, *Pinus halepensis* Mill. and *Quercus ilex* L. were subjected to 3 fertilization treatments (250 kg N, ha^-1^, 250 kg P. ha^-1^, and both) under irrigated and water withholding conditions [2]. The authors found reduced terpenoid emissions by 38% from *P. halepensis* under nutrient supply, independently on water treatment and no effect was noticed for *Q. ilex *monoterpenoid emissions. 

Harley *et al*. (1994) [39], used a full factorial analysis with 3 levels of nitrogen fertilization (NH_4_N0_3_, 12, 8, and 2 mM total nitrogen) and 2 levels of light (300 and 800 µmol, m^-2^, s^-1^) applied to Velvet Bean leaves. They reported increase in isoprene emissions as leaf nitrogen concentration increased, irrespective of light treatment, although this result was most pronounced at high PFD (photon flux density). A 5-fold increase of isoprene was found for a 3-fold increase in leaf nitrogen content. Likewise, isoprene emissions of aspen (*Populus tremuloides *Michaux.) and oak (*Quercus alba* L.) were positively correlated to leaf nitrogen concentrations, both in shade and sun grown plants (PFD reduced by 80% and 2200 µmol m^-2 ^s^-1 ^respectively) [20]) Furthermore, for any given leaf nitrogen concentration (expressed per unit leaf mass), sun leaves emitted greater isoprene rates than shade leaves. 

Fertilization with NH_4_NO_3_ did not alter terpenoid concentration in *Pinus sylvestris* L. and *Picea abies* Karts., either under high or ambient O_3_ concentrations [22] (Fig. (**2**)). Camphor was the exception, since its concentration increased in the fertilized trees (48 and 121 Kg.ha^-1^) when O_3_ concentration increased (by 1.7-fold the O_3_ concentration in the control plots. Characterizing the environmental conditions other than fertilization, before and during the sampling campaigns may hence provide an explanation to the observed results. 

## 4 IMPACT OF FERTILIZERS ON TERPENOID DIVERSITY 

Several arguments considerd that excess fertilization lead plants to store and release a different bouquet of terpenoids. First, the amazing diversity of terpenoids, and more generally all secondary metabolites, are considered as the result of selection pressures, both abiotic and biotic, that concomitantly operate on plant species. Second, changes in some abiotic conditions induce the formation of terpenoids. For example, O_3_ fumigation induced, after 23 h, sesquiterpenoid emissions (b-elemene, aromadendrene, a-humullene) from tobacco plants [40]. Continuous light induced constant emissions of the sesquiterpenoid alpha-copaene in *Lycopersicon esculentum* Mill [41]. Sesquiterpenoid emissions were strongly inhibited after 7 days of water withholding in *R. officinalis*, thus altering terpenoid emission composition [42]. 

Nevertheless, experimental data indicate that different ranges of soil nutrient concentrations do not induce the appearance of new terpenoids, whether these are induced or constitutive [36]. In some cases, nutrients may involve changes in the relative ratios among the different compounds (quality) emitted or stored [43]. Excessive nutrient supply has also been found to strongly limit the concentration of a given terpenoid. Thus, at 470 kg.ha^-1^ NH_4_NO_3_ and 140 Kg.ha^-1^ potassium sulphate, p-cymene concentration in the essential oil of caraway (*Carum carvi* L.) fruit was only detected in trace amounts in comparison with unfertilized treatments and lower doses [44]. 

## 5 FUTURE DIRECTIONS TO STUDY THE CONSEQUENCES OF NITROGEN FERTILIZATION ON TERPENOID PRODUCTION 

Literature of nutrient impact on terpenoid production in natural plants has mainly sought to determine the impact of mineral fertilization, especially NH_4_NO_3. _While most studies point out a positive effect of NH_4_NO_3 _on terpenoid emissions, results on terpenoid storage are little conclusive, in agreement with the investigations that have tested the impact of nitrogen fertilization on essential oils (rich in terpenoid-like compounds) [45]. 

We contend that part of the seemingly discrepancies between different investigators could probably be clarified if the following parameters were taken into account (i) anatomical, physiological and biochemical analyses, since changes in some of these properties (e.g. increase in gland size and number) could counteract modifications of physiological processes (e.g. decrease in photosynthesis) (ii) measurements performed over the seasonal cycle since growth requirements vary over the year and consequently, trade-offs between primary and secondary metabolism could be accordingly adjusted, (iii) knowledge of the optimal, intermediate and limiting nutrient requirements for the studied species in order to reasonably compare results with the GDBH (iv) complementary data on irrigation requirements and other abiotic conditions such as possible pollution and CO_2_ concentrations, which may induce some response in the terpenoid metabolism [46] (v) measurement of appropriate indicators of water and nutrient soil status. Different nutrient forms could not be equally correlated to terpenoid production. In case of soil nitrogen, total nitrogen, NH4^+^ and NO_3_^-^ could be evaluated. Since losses of NH4^+^ occur mainly by volatilization, when H^+^ is removed from NH4^+^ by another ion such as hydroxyl (OH^-^), collected soil samples must immediately be frozen in order to limit such losses. Also, the irrigation frequency and volume should carefully be chosen and measured. Most studies dealing with fertilization regularly irrigate the plants in order to limit any interaction with water stress, which is known to deeply affect terpenoid production. Major losses of NO_3_^- ^can however occur, especially in coarse-textured soils where water percolates freely, since this nitrate form of nitrogen is very soluble and leaches easily.

Also, both soil and leaf nutrient status should be checked since increases in soil nutrients may not directly increase tissue nutrient nutrition [47], a phenomenon that can occur under important runoff. Kainulainen *et al*. (2000) [22] reported unexpected low nutrient concentrations (phosphorus and nitrogen) in leaves of fertilized pots which were imputed to the heavy rain periods during the sampling campaign which probably caused leaching of nutrients from the pots. It can also occur if nutrient supply significantly enhances leaf biomass thus diluting leaf nutrient concentration, under poor root development or if a nutritional imbalance between nitrogen and other nutrients occurs, some of which may be more limiting than nitrogen [48]. 

Since massive fertilization does not occur as an isolated phenomenon but in areas with high population and economic growth, it co-occurs together with increasing air pollution, water deficiency, high temperatures, as well as land use changes. While the individual effects of these changes have been correlated to terpenoid production and other BVOCs we suggest future research to consider the soil fertilizing effect combined to other global change-related phenomena Fig. (**2**). This should be integrated into future scientific protocols in order to accurately estimate BVOC changes. So far, only a few studies have addressed these interactions as previously shown over this review (drought and soil fertilization [2]; light and soil fertilization [20]). Interaction with other environmental factors, such as light and competition, is also crucial since changes in terpenoid defenses due to fertilization in the field is the result of complex interactions between abiotic and biotic factors and should not be reduced to the knowledge of the effect of each nutrient. For example, fertilization promotes plant productivity (plant height and biomass) increasing shading which strengthens competition among plants. Since both, shading and competition affect the production of terpenoid compounds [49,50], fertilization can indirectly influence carbon-based defense metabolism.

Additionally, while the effect of nutrients through mineral fertilization has largely been addressed, the impact of nutrient addition through organic fertilizers has been poorly tested [51,52]. Organic amendment, usually occurring through compost application, is however massively applied in terrestrial ecosystems since compost dumping has been banned in the European Community generating a renewed interest in studying novel ways for recycling it. Large scale compost application occurs in both agro-ecosystems, to improve their productivity, and naturally degraded ecosystems, to favor their regeneration. 

## Figures and Tables

**Fig. (1):**
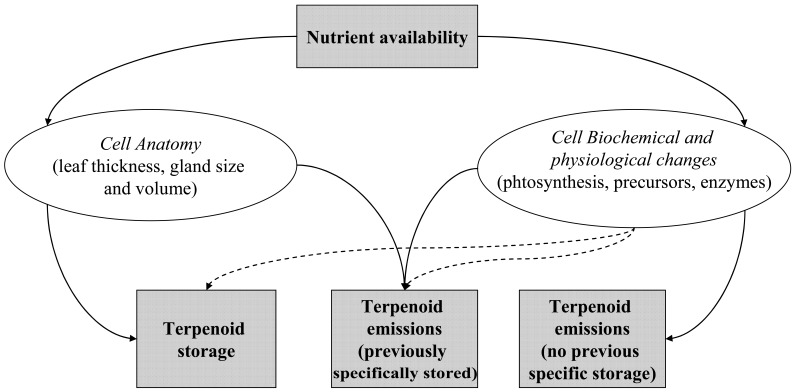
Diagram representing the effect of nutrients on terpenoid storage and emission. Continuous and discontinuous arrows indicate a direct and indirect relationship.

**Fig. (2):**
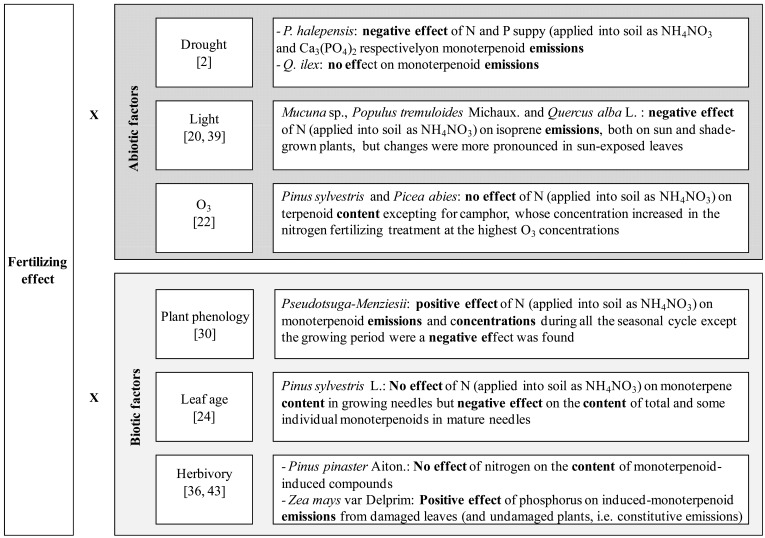
Terpenoid production (storage and emission) as affected by soil fertilization under different biotic and abiotic conditions.

**Table 1.** Terpenoid Concentration in Specific Storage Structures Under Different Soil Nutrient Conditions. Results on Soil Fertilization and Soils Naturally Presenting Differences in their Soil Nutrients are Taken into Account

Reference and Species		Results Reported	Growing Conditions and Period of the Study	Fertilizer: Type and Levels in Soil
***Effect of soils where fertilizers have been applied***				
Close *et al*. [25]	* Eucalyptus globules * * Eucalyptus nitens*	** Positive effect** on terpenoid content in nursery treatment ** No effect** in field conditions	Nursery and field conditions March, April, May, June	* 2 nursery treatments*: 1.25 mg Peters Excel(1) per plant once or twice a week * 2 Post-planting fertilization*: 120 g per plant of (NH_4_)_2_HPO_4_ (diammonium phosphate), N:P:K, 18:46:0 and no fertilizing addition
Kainulainen *et al*. [22]	* Pinus sylvestris* L. * Picea abies* Karts.	** No effect** on monoterpenoid concentration ** Positive effect** on camphor at the highest O_3_ concentrations	Greenhouse conditions 3-and 4-year-old seedlings in 7.5 l plastic pots with sand and fertilized peat Growing season	NH_4_NO_3 _(ammonium nitrate) * Pinus*: 32 and 105 Kg.ha^-1^ * Picea* : 48 and 121 Kg.ha^-1^
McCullough and Kulman [53]	* Pinus banksiana* Lamb.	** Positive effect ** on monoterpenoid concentration on both wildfire and clear cutting sides	Natural conditions 7-11-year-old plants April-May	NH_4_NO_3_ Unfertilized and 140 Kg.ha^-1^
Mihaliak and Lincoln [31]	* Heterotheca subaxillaris *Lam.	** Negative effect** on leaf mono- and sesquiterpenoid content	Growth chamber Seedlings Perlite, vermiculite, and sand (1:1:1)	KNO_3_ and Ca(NO_3_)_2_ Unfertilized, 0.5, 1.5, 5.0 M, and 15.0 mM
Muzika *et al*. [54]	* Abies grandies* (Dougl) Lindl.	** Negative effect** of the higher fertilizing treatment on concentration of 4 terpenoids out of 10 compounds quantified ** No effect** of lower fertilization treatment	Greenhouse conditions Seedlings	NH_4_^+^ and NO_3_^-^ at 2 rates: 224 and 448 Kg.ha^-1^
Powell and Raffa [55]	*Larix laricina *Mélèze laricin.	**Positive effect** on b-pinene concentrations in July	Greenhouse conditions 2 year-old seedlings 18 l liter pots containing washed silica sand and peat (16:1) May, June, July Plants were watered once or twice daily	Osmocote 19-6-12 0.5, 10.0 and 50.0 g
Sampedro *et al.* [36]	*Pinus pinaster* Aiton.	**No effect** on monoterpenoid concentration whether induced or constitutive	Greenhouse conditions Seedlings 2.0 l pots containing sterilized perlite August Daily watering by subirrigation	Phosphorus at two rates: 20 and 2 mg .l ^-1^
***Effect of soils naturally differing in their nutrient content***				
Barnola and Cedeño [56]	* Pinus caribea * Morelet.	** Higher terpenoid concentrations ** in siliceous soils	Field study Adult trees, mature needles Dry season	Calcareous and siliceous soils
King *et al*. [28]	* Eucalyptus polybractea* R. Braker.	** Slight positive** **effect** of leaf nitrogen on terpenoid content in oil glands ** No effect** of leaf phosphorus on terpenoid	Field study Adult trees June	Altitude and slope transects in forest ecosystem
Moretti *et al.* [57]	* Rosmarinus officinalis *L.	** Positive ** effect of soil phosphorus and nitrogen soil nutrients (calcareous soils) on 1,8-cineol amounts	Field study June	Calcareous and siliceous soils
Ormeño *et al.* [58]	*Cistus albidus L.* *Rosmarinus officinalis L.* *Pinus halepensis Mill.*	*C. albidus : * **Negative effect** of soil available phosphorus and total nitrogen on monoterpenoid emissions (calcareous soils) *R. officinalis *and *P. halepensis: ***Positive effect **of soil available phosphorus and total nitrogen on monoterpenoid emissions (calcareous soils) **No effect** of soil nutrients on sesquiterpenoids.	Field study Adult trees, mature and young needles pooled together July	Calcareous and siliceous soils
Robles and Garzino [59]	*Cistus albidus* L.	**Higher concentrations** of many terpenoids in calcareous soils-growing plants (e.g. Allo-aromadendrene, β-caryophyllene) **Higher concentrations **of many terpenoids in siliceous soils (e.g. β-bourbonene)	Field study Mature plants Dormancy period	Calcareous and siliceous soils
Flamini *et al*. [60]	* Myrtus communis * L.	** Higher concentrations** of α-pinene and δ-limonene in calcareous soils-growing plants ** Higher concentrations** of linalool in siliceous soils-growing plants	Field study Mature plants Fructification period	Calcareous and siliceous soils

N:P:K (20:2.2:6.6) solution concentration 1 g.l^-1^
